# The spatial distribution of ERGs reflecting luminance and L-/M-cone-opponent signals

**DOI:** 10.1007/s10633-020-09807-7

**Published:** 2021-01-28

**Authors:** Kallene Summer Vidal, Avinash J. Aher, Dora Fix Ventura, Jan Kremers

**Affiliations:** 1 Department of Ophthalmology, University Hospital Erlangen, Schwabachanlage 6, 91054 Erlangen, Germany; 2 Deparment of Anatomy II, FAU Erlangen-Nürnberg, Erlangen, Germany; 3 School of Optometry and Vision Science, University of Bradford, Bradford, UK; 4 Laboratory of Vision, Institute of Psychology, University of São Paulo, São Paulo, Brazil

**Keywords:** Electroretinography, Spatial distribution, Luminance, Cone opponency, Post-receptoral pathways

## Abstract

**Purpose:**

To study the spatial retinal distribution of electroretinographic (ERG) responses that reflect signals in the L-/M-cone-opponent and luminance post-receptoral pathways.

**Methods:**

ERG recordings to heterochromatic stimuli (sinusoidal counter-phase modulation of red and green LED light sources) were performed, while varying fractions of red and green modulation. Two temporal frequencies of the stimuli were employed: 12 Hz to record ERGs that reflect L-/M-cone-opponent signal and 36 Hz for recording ERG signals sensitive to stimulus luminance. Stimuli were about 20° in diameter and projected on various retinal locations: the fovea and four eccentricities (10°, 19°, 28° and 35°), each presented nasally, temporally, inferiorly and superiorly from the fovea.

**Results:**

The 36 Hz stimuli elicited responses that strongly varied with red fraction and were minimal at iso-luminance. Moreover, response phases changed abruptly at the minimum by 180°. In contrast, the responses to the 12 Hz stimuli had amplitudes and phases that changed more gradually with red fraction. The 36 Hz response amplitudes were maximal close to the fovea and sharply decreased with increasing distance from the fovea. The responses to 12 Hz stimuli were more broadly distributed across the retina.

**Conclusions:**

In the present study, it was found that retinal eccentricity and direction from the fovea have distinct effects on ERGs reflecting different post-receptoral mechanisms. The results are in accord with previous findings that ERGs to 12 Hz stimuli are predominantly determined by the red–green chromatic content of the stimuli, thus reflecting activation in the L-/M-cone-opponent pathway, while responses to 36 Hz stimuli manifest post-receptoral luminance-dependent activation. We found that the response in the cone-opponent pathway is broadly comparable across the retina; in comparison, response amplitude of the luminance pathway strongly depends on retinal stimulus position.

## Introduction

Electroretinography (ERG) is an important tool used in clinical and basic research to study the integrity of the retina and the physiology of cellular circuitries that are involved in visual processing [1, 2]. Until recently, with a few exceptions (e.g., Sperling [3]), there was limited correlation between cone signal characteristics in the ERG and in retino-geniculate pathways. However, in the past 12 years, ERG responses to continuous stimuli that contained luminance and red-green chromatic modulations (e.g., L- and M-cone isolating or heterochromatic stimuli) were found to reflect cone-opponent and luminance activity, probably reflecting activity in the parvo- and magnocellular pathways, respectively [4–8]. Using sinusoidal stimuli, cone-opponent signals were found at temporal frequencies below 16 Hz, whereas luminance-reflecting signals were obtained at frequencies above 30 Hz.

Subsequently, it was found that the ERGs reflecting activity in the two pathways have different response characteristics when using circular and annular stimuli of different sizes [9, 10]. In these studies, it was found that luminance-reflecting ERGs were positively correlated with stimulus size, whereas ERGs that reflect cone-opponent responses surprisingly were fairly constant for a large variety of stimulus sizes. Interestingly, this was found for circular and annular stimuli [9, 10]. These results suggest that cone-opponent and luminance-reflecting ERG signals have different spatial distributions. A direct measurement of the spatial distributions of these ERGs is, however, lacking. It is the purpose of the present study to investigate the spatial signal distribution by recording ERGs to 12 and 36 Hz heterochromatic modulation of red and green light sources (to obtain ERGs that reflect cone-opponent and luminance responses, respectively) at different retinal locations.

We performed extensive measurements with three subjects including a relatively large number of heterochromatic modulations. We then performed the measurements using a subset of heterochromatic modulations in seven additional subjects.

## Material and methods

### Subjects

Ten healthy volunteers (6 females, 4 males; age between 28 and 58 years, mean [± SD]: 35.7 ± 8.2 years) participated in this study. The study was approved by the ethics committee of the University Hospital Erlangen, Friedrich-Alexander University Erlangen-Nürnberg (# 329_12B) and adhered to the tenets of the Declaration of Helsinki. Subjects were informed and gave their signed consent prior to the experiments. All subjects had normal color vision as established with an anomaloscope (Oculus Optikgeräte GmbH, Wetzlar, Germany) and underwent a complete ophthalmological evaluation including refraction, best-corrected visual acuity, slit-lamp biomicroscopy, intraocular pressure measurements, gonioscopy, and dilated funduscopic examination.

### ERGs measurements

ERGs were recorded from the right eyes of the subjects which were dilated using a drop of 0.5% tropicamide (Pharma Stulln GmbH, Germany) prior to recording. The left eyes were occluded using an eyepatch during the recordings. A fiber electrode, placed over the lower conjunctiva, served as the active electrode. After cleaning the skin with NuPrep® abrasive gel, two gold cup electrodes, filled with electrode paste (DO weaver & Co.), one placed on the forehead and one at the ipsilateral temple, served as ground and reference electrodes, respectively. The impedance of the active and reference electrodes was below 5 KΩ. The signals were amplified 100,000 times, band-pass-filtered between 1 and 300 Hz, and sampled at 2048 Hz. The ERG responses from 80 sweeps, each lasting 1 s, were averaged. The first 2 s of recording time after commencement of the stimulation were disregarded to avoid onset artifacts.

### Visual stimuli

Heterochromatic flicker stimuli were created using a Ganzfeld bowl (Q450SC, Roland Consult, Germany) controlled by the RETIport (Roland Consult) system. Circular stimuli with 20° diameter were created with black cardboard stops positioned 3 cm in front of the subjects' eyes. The size of the hole was 1.06 cm in diameter. The subjects were asked to fixate a red LED at the back of the Ganzfeld bowl at about 40 cm distance from the eye. As a result, the edges of the stimulus were strongly blurred and the luminance profile was approximately Gaussian with the stimulus size being the size of the Gaussian at half maximal luminance. The stimulus’ luminance was 100 cd/m^2^ at the middle of the stimulus as measured with CAS140 spectro-radiometer connected to a TOP 200 device (Instrument Systems, Germany) at the position of the eye and focused at the back of the Ganzfeld bowl. We checked that the stimulus on the retina was indeed spatially restricted by placing a lens (*f* = 17 mm.) at the position of the pupil and measuring the size of the projection at about 15 mm. distance from the lens. We therefore conclude that, although the retinal images of the stimuli did not have sharp edges, they were spatially restricted and therefore were appropriate for the present study.

**Fig. 1:**
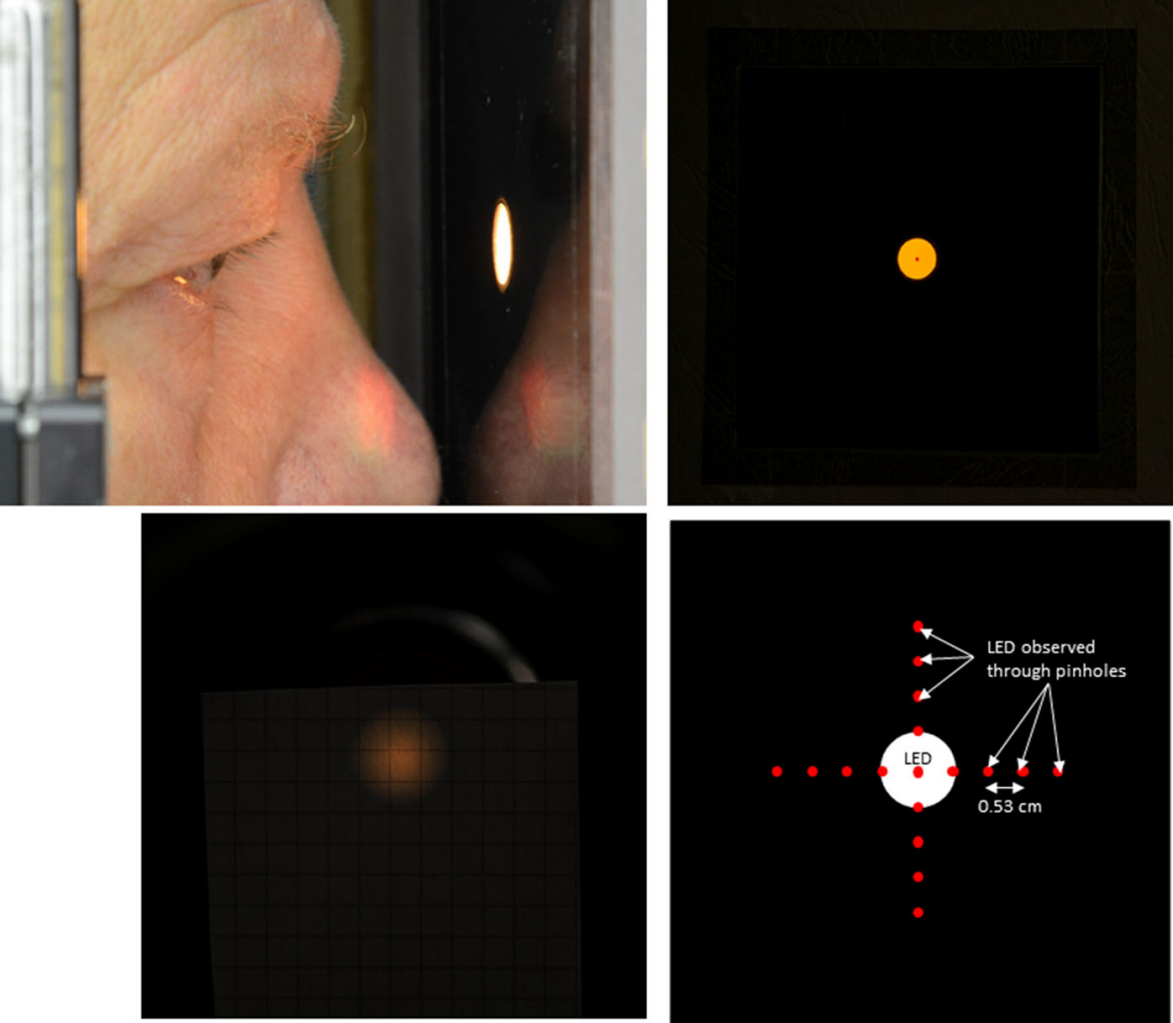
Description of the configuration of the experimental setup. The upper left photograph displays the position of the stop in the cardboard mask relative to the eye of the subject. The upper right photograph is a view of the cardboard stop at about a meter distance with the fixation spot in the center. The lower right plot is a cartoon of the positions of the fixation spots. When the spot is on the card board, they were observed through pinholes punched into the cardboard. The picture in the lower left is a photograph of the image of the stimulus at about 15 mm distance from a lens (*f* = 17 mm.) positioned at about 30 mm from the card board

The stimuli were positioned at the center (fovea) and at 12 additional positions. This was achieved by asking the subjects to position the stimulus such that the fixation spot could be observed in the center of the stimulus (for a foveal stimulus) at the edge of the spot (for a stimulus with 10° eccentricity) and through small pinholes that were punched in the cardboard at one, two and three radii (in cm) from that edge (for further eccentric stimuli). This procedure was performed along the superior and inferior vertical meridian as well as along the nasal and temporal horizontal meridian. We calculated that the eccentricities of the stimuli were at 0°, 10°, 19°, 28° and 35°. The more eccentric stimuli appeared increasingly elliptical through trigonometric distortion. As a result, we estimated that the actual stimulus areas were about 0.97. 0.89, 0.79 and 0.68 times the foveal stimulus at 10°, 19°, 28° and 35° eccentricities, respectively. We measured the luminance at the center of the stimulus at various angles and did not find a systematic or significant change. See Fig. 1 for illustrations of the setup.

**Fig. 2:**
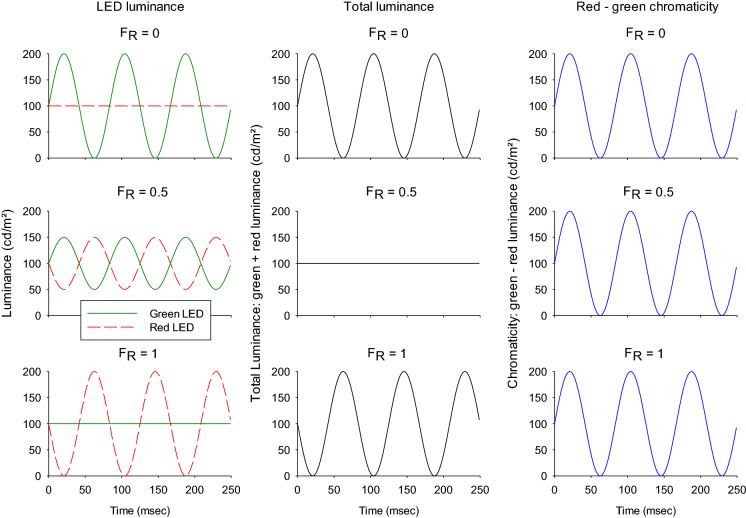
Representation of three different stimulus conditions at 12 Hz temporal frequency (i.e., three cycles at the displayed period of 250 ms): *F*_R_ 0, 0.5 and 1. *F*_R_ = 0 indicates that the red light-emitting diode (LED) was constant and the green LED was modulated with 100% contrast. *F*_R_ = 0.5 indicates that both the red and green LEDs were modulated in counterphase with 50% contrast and, with *F*_R_ = 1, the red LED was modulated with 100% contrast and the green LED was not modulated. The plots in the middle show the resulting luminance modulation (assuming V_λ_ spectral sensitivity) of the three conditions obtained by addition of the luminances in each LED. The luminance modulation is minimal at *F*_R_ 0.5. At the minimum, the luminance phase changes by 180°. The output of a red-green chromatic system (after subtraction of the luminances in the two LEDs) is displayed in the right column. Here the amplitudes and phases do not change with *F*_R_ value

The outputs of red (with Michelson contrast C_R_) and green (with Michelson contrast C_G_) light-emitting diodes (LEDs) were modulated sinusoidally in counter-phase around a mean luminance. Stimulus conditions with varying red and green contrasts were employed while keeping the total contrast (C_R_ + C_G_) constant at 100%. The stimulus conditions were quantified by the red contrast fraction [*F*_R_ = C_R_/(C_R_ + C_G_)]. For example, for *F*_R_ = 0, C_G_ was 100% and red LED was not modulated (i.e., C_R_ = 0%). At *F*_R_ = 1 C_R_ was 100% and C_G_ was 0%. At *F*_R_ = 0.5, both red and green LEDs were modulated with 50% contrast (C_R_ = C_G_ = 50%; see Fig. 2). In three subjects (authors KVS, AJA and JK), nine conditions at 36 Hz (*F*_R_ 0, 0.2, 0.3, 0.35, 0.4, 0.5, 0.6, 0,8 and 1) were employed. At 12 Hz, measurements with *F*_R_ 0, 0.2, 0.4, 0.6, 0.8 and 1 were performed. These measurements lasted in total about 7 h per subject and were performed in several sessions. In the measurements with the remaining seven subjects we used a subset of *F*_R_ values 0 and 1 at 12 and 36 Hz to obtain more data for reliable statistical analysis. The measurements lasted about 1.5 h and were performed in one session.

### Data analysis

The signals were analyzed using fast Fourier transform (FFT) with a MATLAB program (R2013b, MathWorks, Natick, MA) written by author JK. The amplitudes and phases of the first harmonic (fundamental) components were used to describe the responses. The amplitude and phase data were disregarded when the signal-to-noise ratio (quantified by the ratio of the fundamental component and the average of the component amplitudes at adjacent frequencies; e.g., values at 11 and 13 Hz for 12 Hz responses) was smaller than 2.

## Results

**Fig. 3:**
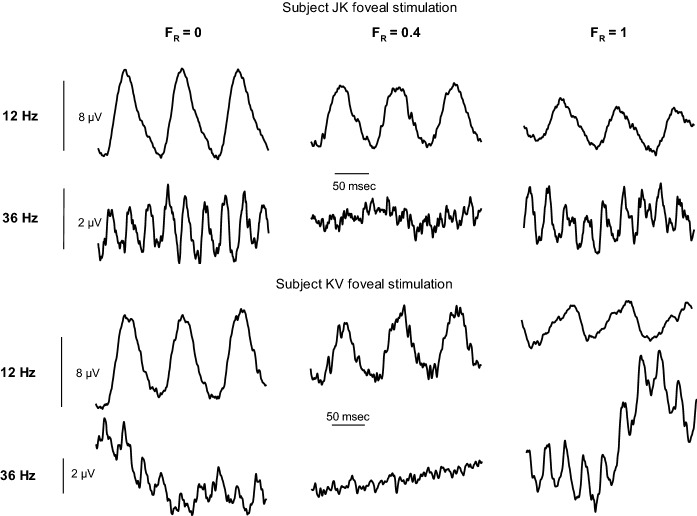
Averaged original responses measured in two subjects for foveal stimuli at 12 and 36 Hz and for *F*_R_ values 0, 0.4 and 1.0. The responses were averaged for epochs of 250 ms

Figure 3 displays the original responses measured in two subjects for foveal stimulation at *F*_R_ values 0, 0.4 and 1.0 and at 12 and 36 Hz. The responses at 12 and 36 Hz clearly have different characteristics as a function of *F*_R_: While the 12 Hz responses gradually decrease with increasing *F*_R_, the 36 Hz responses are clearly minimal at *F*_R_ = 0.4 (which is the isoluminance condition for most trichromats). Furthermore, the timing of the responses is similar at 12 Hz, but close inspection of the 36 Hz responses shows that the responses at *F*_R_ values 0 and 1 are close to counterphase relative to each other.

**Fig. 4:**
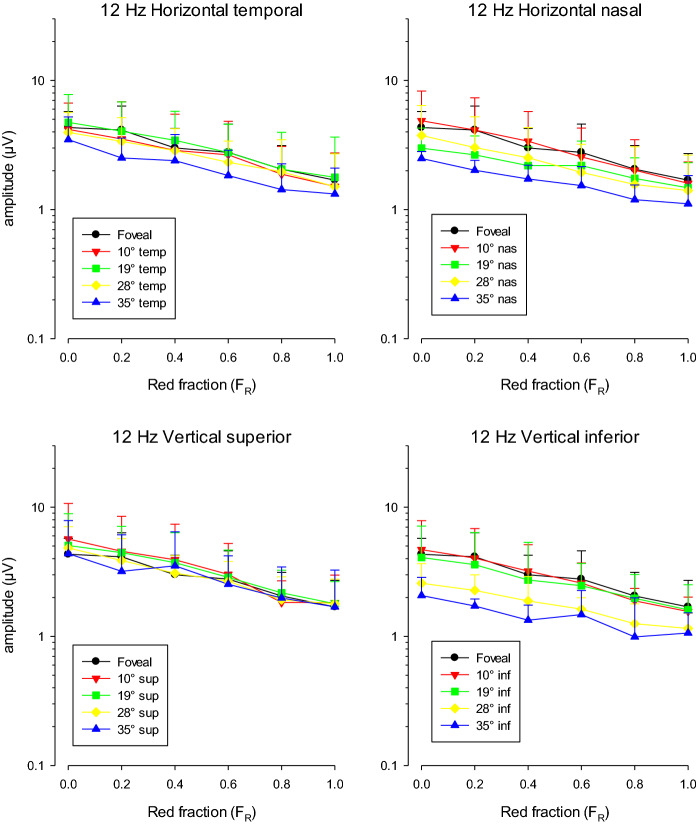
ERG response amplitude (in μV) to 12 Hz stimuli as a function of *F*_R_. The means (+ 2 SD) of the responses of three subjects are displayed. The upper plots show response amplitude for stimuli on the horizontal meridian. The lower plots show those elicited by stimuli on the vertical meridian. The data for the different stimulus locations are shown separately

Figure 4 shows the mean (+ 2 SD) amplitudes of the 1^st^ harmonic response components to 12 Hz stimuli (on a logarithmic scale) as a function of *F*_R_. The responses were measured in three subjects. The upper plots show the data for the measurements with the stimuli located on the horizontal meridian (left plot for temporally located stimuli; right plot for nasally located stimuli). The lower plots show the data for measurements with stimuli on the vertical meridian (left: superior; right: inferior).

The amplitude depended on *F*_R_ in a similar manner for all stimulus positions with a gradual decrease with increasing *F*_R_ values. Although these data are obtained from only three subjects, they are preliminary observations about the response amplitudes at different stimulus positions. To enable an estimate of the statistic value of these observations, the error bars in the amplitude plots represent 2 SD. Similar absolute amplitudes were found for the foveal stimuli and those at 10° and 19° eccentricities. The responses to some stimuli at 28° and 35° eccentricity were smaller than those to the centrally located stimulus. For all stimuli located superior to the fovea (lower left plot), the response amplitudes were similar to each other and to the foveal response, whereas there was an amplitude decrease with distance to the fovea for the inferior stimuli (lower right plot). To be able to draw firmer conclusions about the relationship between response amplitude and stimulus position, we performed measurements in additional subjects using a subset of *F*_R_ values (see below).

**Fig. 5:**
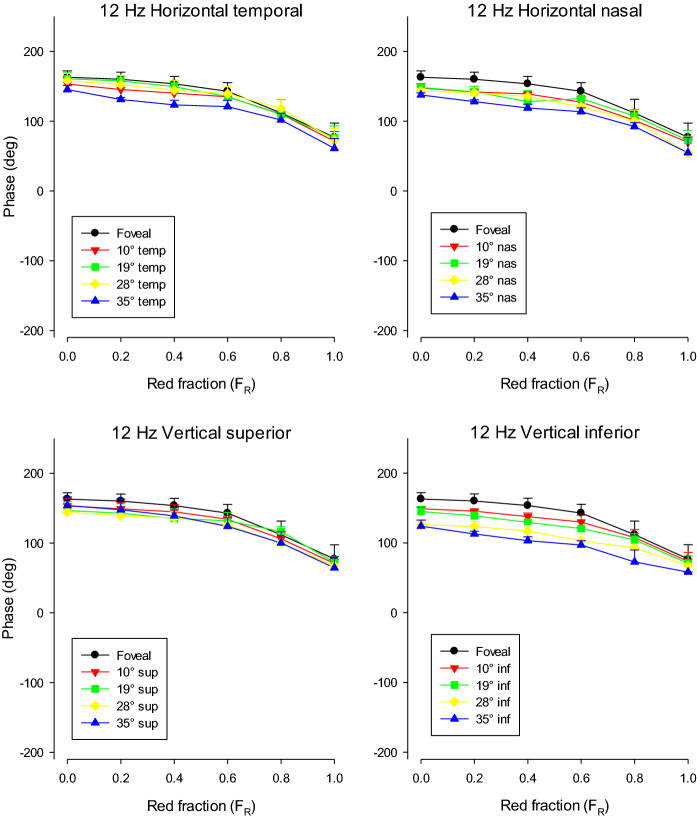
ERG response phase (in °) to 12 Hz stimuli as a function of *F*_R_. The means (+ 1 SD) of the responses of three subjects are displayed. The upper plots show the results for stimuli on the horizontal meridian. The lower plots show those elicited by stimuli on the vertical meridian. The data for the different stimulus locations are shown separately

Figure 5 displays the response phase as a function of *F*_R_ for the different stimulus positions. Please note the linear ordinate in the phase plots. The phases changed by about 20° between *F*_R_ = 0 and 0.6 (cf. Figure 1). Between *F*_R_ values 0.6 and 1, the phase change was between 30° and 40°. This phase relationship seemed to be similar for all stimulus positions.

**Fig. 6:**
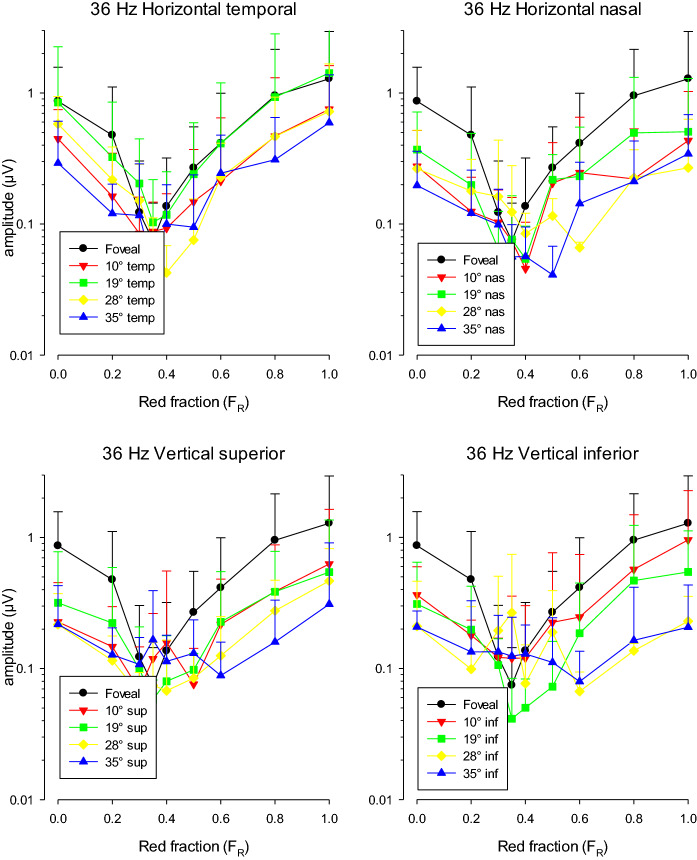
ERG response amplitude (in μV) to 36 Hz stimuli as a function of *F*_R_. The means (+ 2 SD) of the responses of three subjects are displayed. To enable a direct comparison, the ordinate has the same scale range as in Fig. 4. The upper plots show the response amplitude for stimuli on the horizontal meridian. The lower plots show those elicited by stimuli on the vertical meridian. The data for the different stimulus locations are shown separately

Figure 6 shows the mean response amplitudes for 36 Hz stimuli. The ordinate of these plots has the same (logarithmic) scale range as the plots shown in Fig. 4 (enabling a comparison of the data), but is shifted by a factor of 10 towards lower amplitudes because the 36 Hz responses were smaller than those to the 12 Hz stimuli. The 36 Hz responses were much smaller when using 20° diameter stimuli compared to full field stimulation (contrary to responses to 12 Hz stimuli where the responses to full field and 20° stimuli had similar amplitudes). As a result, the data were also noisier than the 12 Hz responses.

The amplitudes strongly depended on *F*_R_ showing a minimum at *F*_R_ values of about 0.4, indicating that the responses reflected luminance activity because the minimal luminance content in the stimulus is close to the minimum for this condition (cf. Fig. 1, middle column). Furthermore, it can be seen that the response amplitudes decreased strongly with increasing stimulus eccentricity in all four directions. Again, these preliminary observations are obtained from only three subjects. We will return to this issue in more detail (including a statistical analysis) when describing the data in all subjects.

**Fig. 7:**
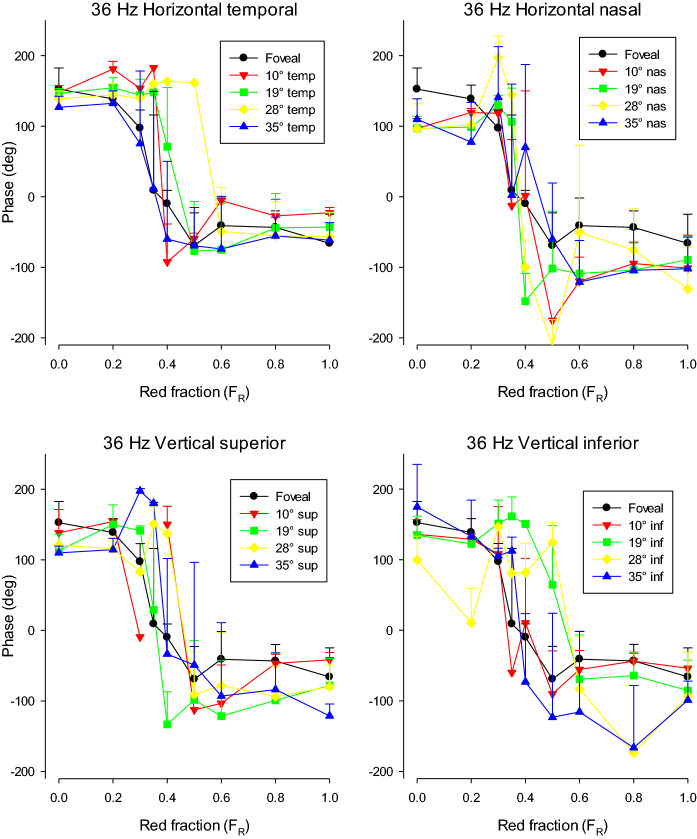
ERG response phase (in °) to 36 Hz stimuli as a function of *F*_R_. The means (+ SD) of the responses of three subjects are displayed. To enable a direct comparison, the ordinate has the same scale as in Fig. 5. The upper plots show the results for stimuli on the horizontal meridian. The lower plots show those elicited by stimuli on the vertical meridian. The data for the different stimulus locations are shown separately

At the minima, the 36 Hz response phase (shown in Fig. 7) changed strongly (close to 180°). This phase relationship was observed for all stimulus positions. The phase data were more variable than in the 12 Hz data (cf. Fig. 5) because the responses were smaller and noisier. As mentioned in the methods section, the data were disregarded if the SNR was below 2..

**Fig. 8:**
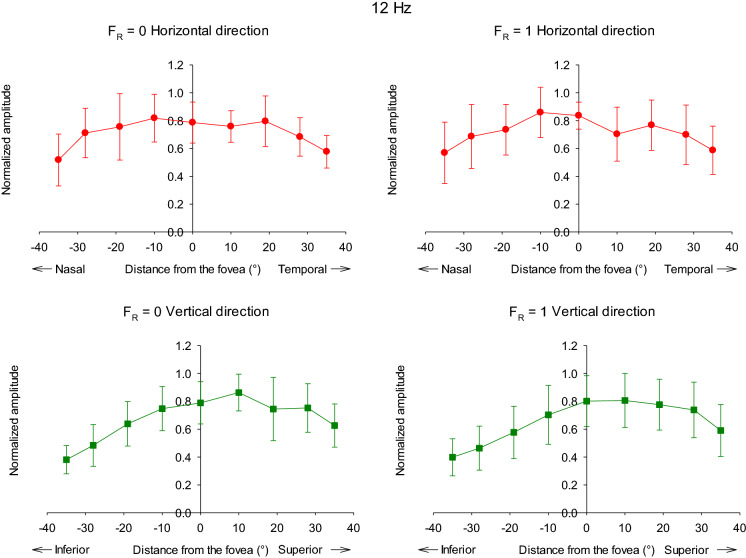
Averaged response amplitude (μV) of ERGs to 12 Hz versus stimulus eccentricity obtained from all ten observers. The amplitudes were normalized to the largest amplitude values for each individual in the horizontal (upper plots) and vertical regions (lower plots). The left plots show the data for *F*_R_ = 0; right plots display the data for the *F*_R_ = 1 conditions

The responses were measured in seven additional subjects for *F*_R_ = 0 and 1.0. Figure 8 shows the averaged 12 Hz response amplitudes obtained from ten subjects as a function of retinal eccentricity plotted separately for *F*_R_ = 0 (left plots) and for *F*_R_ = 1 (right plots) and for stimuli along the horizontal (upper plots) and vertical (lower plots) meridians. The amplitudes were normalized for each subject to the maximal individual response separately for the two *F*_R_ values and for measurements along the horizontal and the vertical meridians. The response distributions were relatively broad, indicating that the response amplitudes did not change strongly as a function of position (as can also be observed in the data of the three subjects with more extensive data shown in Fig. 4).

The response amplitudes were not symmetrically distributed around the fovea. Furthermore, the asymmetry is particularly clear for the vertical locations with smaller responses for stimuli that are projected on the inferior part of the retina. The asymmetry along the vertical axis is statistically significant. (The data were not normally distributed; Mann–Whitney rank-sum test: p < 0.001; only the responses with *F*_R_ = 1 were evaluated because this condition is close to the silent substitution condition for rods and thus rod intrusion is expected to be absent or very small.)

**Fig. 9:**
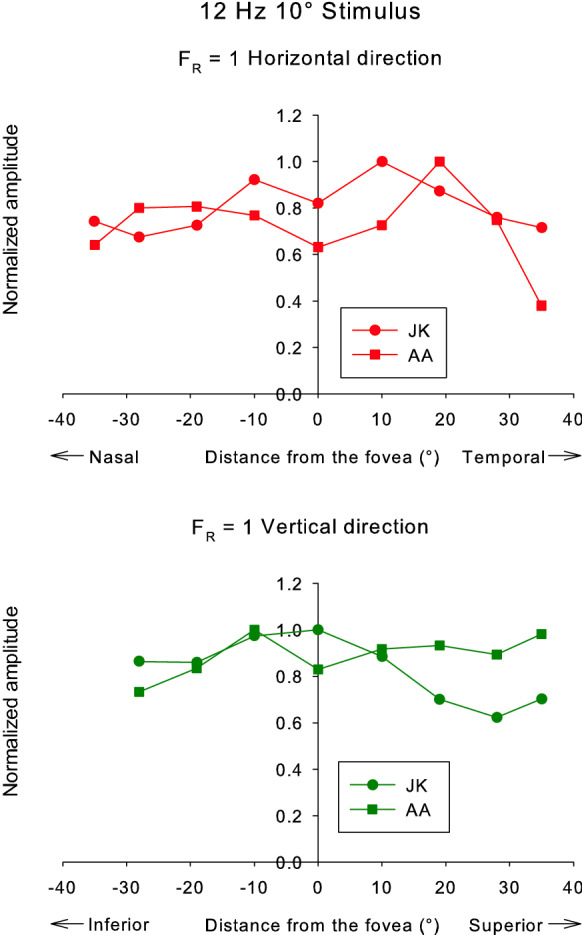
Individual response amplitudes (μV) of ERGs to 12 Hz stimuli versus stimulus eccentricity for a 10° stimulus obtained from two subjects. The amplitudes were normalized to the largest amplitude values for each individual in the horizontal (upper plot) and vertical regions (lower plot). In these measurements, only *F*_R_ = 1 conditions were used. Although the stimulus is smaller than the one used in the main experiment, the distribution is still broad, similar to the data shown in Fig. 8

We repeated the measurements in two subjects with a 10° stimulus at *F*_R_ = 1. The measurements at the 35° inferior position were discarded because the subjects reported that they could not see this stimulus and the responses were at noise level. This observation may also be the cause of the smaller responses to 20° stimuli at this position. The remaining results were in agreement with those described above for the 20° stimulus (see Fig. 9).

**Fig. 10:**
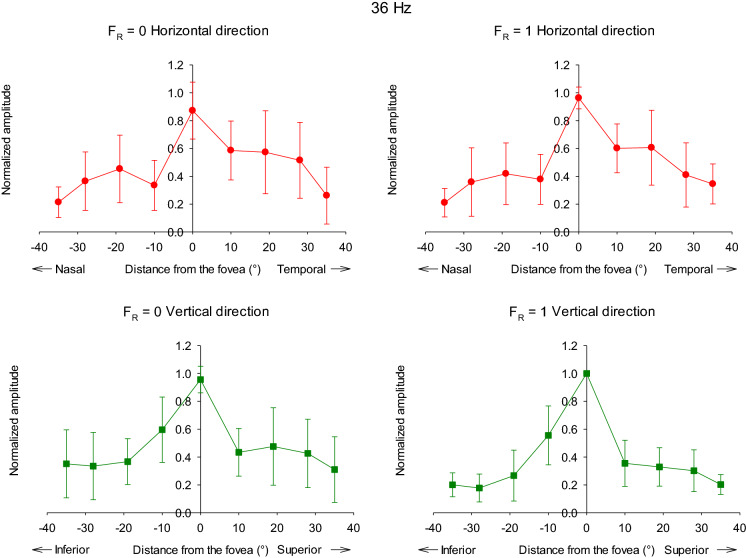
Averaged response amplitude (μV) of ERGs to 36 Hz as a function of stimulus eccentricity. The averages are from all ten observers and the amplitudes were normalized to the largest amplitude values for each individual in the horizontal (upper plots) and vertical regions (lower plots). The left plots show the data for *F*_R_ = 0; right plots display the data for the *F*_R_ = 1 conditions

Figure 10 displays the averaged normalized amplitudes for the 36 Hz responses. Again the results for *F*_R_ = 0 and 1 conditions were very similar. In contrast with the 12 Hz data, the responses were maximally for the foveal stimuli. Nearly all subjects displayed maximal responses for the central stimuli. Furthermore, the response amplitudes decreased more strongly than the 12 Hz responses with distance to the fovea. While the responses to 12 Hz stimuli at 10° eccentricity were either larger or decreased by maximally 15%, the 36 Hz responses decreased by 30–60% at the same eccentricity. The 36 Hz stimuli elicited responses at 28° eccentricity that were between 60 and 80% smaller than the response to central stimulus. For comparison, the 12 Hz responses at 28° eccentricity were of similar size as those of the central responses or were decreased by about 50% when located in the inferior retina.

Finally, as with the 12 Hz data, an asymmetry was present in the responses to 36 Hz stimuli. However, the asymmetry at 36 Hz was particularly obvious for horizontal stimulus locations with larger responses to stimuli that were located temporally of the fovea. The asymmetry along the horizontal meridian was statistically significant (Mann–Whitney rank-sum test: p = 0.003 when all eccentricities were included; only the responses with *F*_R_ = 1 were evaluated).

## Discussion

The purpose of the present study was to describe the spatial response distribution of ERGs reflecting L-/M-opponent and luminance activity. We therefore measured ERGs at different distances from the fovea along the horizontal and vertical meridians. From previous experiments with different stimulus sizes and configurations, we inferred that ERGs at 12 Hz and 36 Hz had different spatial properties [9, 10]. The data of the present study confirmed this.

At 12 Hz, amplitude and phase did not change strongly but gradually as a function of *F*_R_ (see Figs. 4, 5). These findings are in agreement with previous data [5, 10] and indicate that the ERGs reflect cone-opponent activity (Fig. 2; right column). The amplitude (and phase) of responses that exclusively reflect cone-opponent activity is expected to be constant at all values of *F*_R_ (see also Fig. 2). The gradual amplitude changes are an indication of intrusion of rod signals. This was previously found for spatially restricted stimuli that were created with black cardboard [11]; the intrusion was considerably weaker if a constant luminance in the surround was introduced. Furthermore, rod isolating photopic stimuli elicited responses that were larger with spatially restricted stimuli when compared to full field stimuli. These results indicate that the rod intrusion is caused by stray light stimulating the dark adapted retinal areas around the stimulus. The silent substitution condition for rods is close to *F*_R_ = 1. Thus, rod intrusion is expected to be very small or absent for *F*_R_ = 1.

L-/M-cone-opponent ERGs did not depend strongly on the retinal location of the stimulus. This result corroborates with previous findings that this ERG type neither depended on stimulus form (circular or annular) nor on stimulus size [9, 10]. Only for circular stimuli smaller than 10° in diameter, a correlation between stimulus size and response amplitude was found. Thus, the relationship between stimulus size and response amplitude reaches a plateau, suggesting the presence of a sort of saturating effect [10]. The foveal stimulus used in the present study was 20° in diameter and the stimulus area decreased down to 0.68 times the foveal stimulus at 35° eccentricity. Despite this change in stimulus area, the response amplitudes were relatively constant. Possibly, the same saturating effects therefore may have influenced the results of the current study and are the cause for the broad response amplitude distribution for different retinal locations. We therefore performed a control experiment using 10° in diameter (Fr = 1 at 12 Hz) with similar results compared to the recordings with 20° stimuli (see Fig. 9). These findings indicate that saturation cannot explain the broad distribution and that the cone-opponent mechanism probably genuinely has a broad distribution. Interestingly, ERG responses reflecting cone opponency are relatively independent of stimulus position and stimulus size despite large changes in cone density. This suggests the presence of a compensatory mechanism because the signal output remains relatively constant although the cone signal input may vary considerably. A compensatory mechanism was also proposed to explain the similar responses of cone-opponent ERGs to L- and M-cone isolating stimuli with equal cone contrasts [4, 12] and the similar psychophysical detection sensitivities to L- and M-cone isolating stimuli at low temporal frequencies (where the chromatic pathway is responsible for the task) [13] because L-cone packing density is generally larger than M-cone density in trichromatic subjects. Possibly, the two compensatory mechanisms have identical origins.

The 36 Hz responses to heterochromatic stimuli decrease strongly in amplitude with decreasing stimulus size [10]. As a result, the 36 Hz responses were much smaller with the 20° diameter stimuli compared to full field stimulation (contrary to responses to 12 Hz stimuli where the responses to full field and 20° stimuli had similar amplitudes). The data were therefore noisier than the 12 Hz responses. In addition, the amplitudes strongly depended on *F*_R_ showing a minimum at *F*_R_ values of about 0.4, accompanied by large phase changes. This can be expected from a system with responses that are determined by the luminance content in the stimulus, because this condition is close to the isoluminance point at which the phase of the luminance content changes by 180° (following the green LED at lower *F*_R_ values and following the red LED at higher values; cf. Fig. 2, middle column) [5, 10]. Furthermore, it can be seen that the response amplitudes decreased strongly with increasing stimulus eccentricity in all four directions. However, the amplitude decrease was less strong for temporally located stimuli when compared with those to nasally located stimuli. We propose that the responses for nasally located stimuli are smaller because a substantial part of the stimulus is projected onto the blind spot thereby decreasing the effective stimulus size.

We have found that the luminance-reflecting ERGs were maximal with foveal stimuli and their amplitudes decreased strongly with increasing distance from the fovea. This decrease can partially be explained by the decrease in stimulus size with increasing eccentricity. However, the stimulus area at 10° eccentricity was 0.97 times smaller than the foveal stimulus and probably cannot explain the 30–60% amplitude decrease. It was found before that the luminance-reflecting ERGs are proportional to the number of stimulated cones [14] and cone density was indeed found to be highest in the fovea of humans [15] and other primates [16]. Light adapted flash ERGs likely originate in luminance sensitive retinal mechanisms. Therefore, the spatial distribution of the 36 Hz responses can be expected to be similar to the spatial distribution of the flash ERG measured with focal or multifocal stimuli. Indeed, Miyake [17] found that the focal ERG at 7.5° eccentricity was about half the size of the foveal response. Also in the multifocal ERG, the foveal response is maximal and the response amplitude strongly decreases with increasing eccentricity. Similar to our data, it was found that the response decrease was symmetric in the vertical direction, but that for horizontally located stimuli, the eccentricity dependent decrease was stronger in the nasal than in the temporal direction [18]. A naso-temporal asymmetry in the mfERG was confirmed in subsequent studies (see [19] for an overview). We conclude that the spatial distribution of the 36 Hz responses is probably similar to that of the flash focal ERG and mfERG.

The present study has some limitations that should be mentioned. First, the experimental setup was a compromise to meet the luminance, temporal and spatial requirements. High luminance resolution of the red and green LEDs was required to be able to obtain accurate contrasts. High temporal resolution was necessary to acquire precise sine-wave modulations at the required temporal frequencies. To our knowledge, currently these requirements are only met by Ganzfeld stimulators. To obtain spatially restricted stimuli we had to accept that the retinal image did not have an even luminance distribution (see methods section). Ideally, the stimulus is located on the retinal plane to obtain a sharp retinal image. Currently, monitors and projects are under development using four of five differently colored LEDs as light sources (for high luminance and spectral resolution) and with high temporal and spatial resolution (see, e.g., [20]). These stimulators may be an important improvement for the type of experiments described in the present paper.

A second limitation is that the full set of measurements performed in the present study is quite extensive and last up to several hours (see methods section). Therefore, they can only be obtained from a limited number of subjects. In agreement with the results of previous experiments using similar stimuli [10], the variability in the results was limited so that we are confident that the data are reliable even when obtained from two or three subjects. We used a subset of stimuli for measurements with seven additional subjects and the results confirmed the results obtained with the three subjects.

The different responses in the luminance and chromatic pathways as described in the present study may be useful to create functional maps of these pathways if combined, for instance, with multifocal stimulation. Such functional maps may be useful tools to investigate the post-receptoral activity in different retinal diseases that affect the macular and peripheral regions differently and which have distinct effects on the major retino-geniculate pathways.
